# Body Louse Pathogen Surveillance among Persons Experiencing Homelessness, Canada, 2020–2021

**DOI:** 10.3201/eid3007.231660

**Published:** 2024-07

**Authors:** Carl Boodman, Leslie R. Lindsay, Antonia Dibernardo, Kathy Kisil, Heather Coatsworth, Chris Huynh, Amila Heendeniya, John Schellenberg, Yoav Keynan

**Affiliations:** Institute of Tropical Medicine, Antwerp, Belgium (C. Boodman), University of Antwerp, Belgium (C. Boodman);; University of Manitoba, Winnipeg, Manitoba, Canada (C. Boodman, A. Heendeniya, J. Schellenberg, Y. Keynan);; Public Health Agency of Canada, Winnipeg (L.R. Lindsay, A. Dibernardo, H. Coatsworth, C. Huynh);; Access Downtown, Winnipeg (K. Kisil)

**Keywords:** *Bartonella*, *Pediculus humanus humanus*, bacteria, vector-borne infections, parasites, pediculosis, trench fever, ectoparasitosis, homelessness, body louse, lice, Canada

## Abstract

We analyzed body lice collected from persons experiencing homelessness in Winnipeg, Manitoba, Canada, during 2020–2021 to confirm vector species and ecotype and to identify louseborne pathogens. Of 556 lice analyzed from 7 persons, 17 louse pools (218 lice) from 1 person were positive for the louseborne bacterium *Bartonella quintana*.

In 2020, Canada’s largest cluster of *Bartonella quintana* endocarditis, an infection caused by a louseborne bacterium, was detected among persons experiencing homelessness in Winnipeg, Manitoba, Canada (*1*). Over a 6-month period, 4 people required hospitalization for *B. quintana* endocarditis (*1*). The outbreak triggered a retrospective analysis revealing 11 cases of *B. quintana* in Manitoba in the preceding decade (*2*). In 2022, the first pediatric case of *B. quintana* endocarditis acquired in a high-income country was reported from Manitoba (*3*). Prior to the Manitoba outbreak, only 3 cases of *B. quintana* infection were detected in Canada (*4*).

*B. quintana* is a fastidious gram-negative bacillus transmitted through the feces of infected body lice, *Pediculus humanus humanus* (*5*). The bacterium was first detected during World War I as the cause of trench fever and was later determined to cause bacteremia, endocarditis, and bacillary angiomatosis (*5*). *B. quintana* enters the bloodstream through broken skin (*5*).

Body lice and head lice are morphotypes of a single species, *Pediculus humanus* (*6*). Unlike head lice, body lice live in clothing, intermittently moving to the skin to feed on blood (*5*). Body lice are traditionally known to transmit 3 pathogens: *B. quintana, Rickettsia prowazekii* (epidemic typhus), and *Borrelia recurrentis* (louseborne relapsing fever) (*5*). Whereas they are not typically louseborne, *Coxiella burnetii* and *Acinetobacter* spp. have been detected in body lice (*7*). Body louse infestation is associated with poverty, experiencing homelessness, and an inability to wash and change clothing.

The possibility that body lice–infested persons from Winnipeg could be exposed to louseborne pathogens is unknown. In this article, we discuss what louseborne pathogens were found in Winnipeg body lice and the difference in pathogen real-time PCR cycle threshold (Ct) values according to louse instar and sex. This study was approved by the University of Manitoba and multiple other institutional ethics review boards (Appendix).

## The Study

We collected ectoparasites from the clothing of participants in inner city Winnipeg. We separated ectoparasites from the same person into pools based on instar and sex. We pooled ectoparasites from the first and second instars but tested those from the third and fourth instars separately. We tested ectoparasites positive for *B. quintana* from the fourth instar in separate pools of male and female parasites. We decontaminated ectoparasite pools by using 70% ethanol and homogenized them by using a copper clad bead beater. We then extracted DNA by using the DNeasy 96 kit (QIAGEN, https://www.qiagen.com). We identified vector species, louse morphotype, and pathogens by using real-time PCR (Appendix). We used cytochrome b genes to identify louse species and Phum_PHUM540560 genes to identify ecotype (*8*). We identified pathogens by using the following targets: ITS3, *Bartonella* genus; *yopP* and *fabB*, *B. quintana*; *ompB*, *Rickettsia prowazekii*; IS1111a, *Coxiella burnetii*; and *rpoB*, *Acinetobacter* spp. We conducted statistical analysis by using Mann-Whitney U and Kruskal-Wallis tests (Bonferroni correction, post-hoc Dunn test) to compare groups of Ct values (Appendix). We considered values of p<0.05 significant.

**Figure 1:**
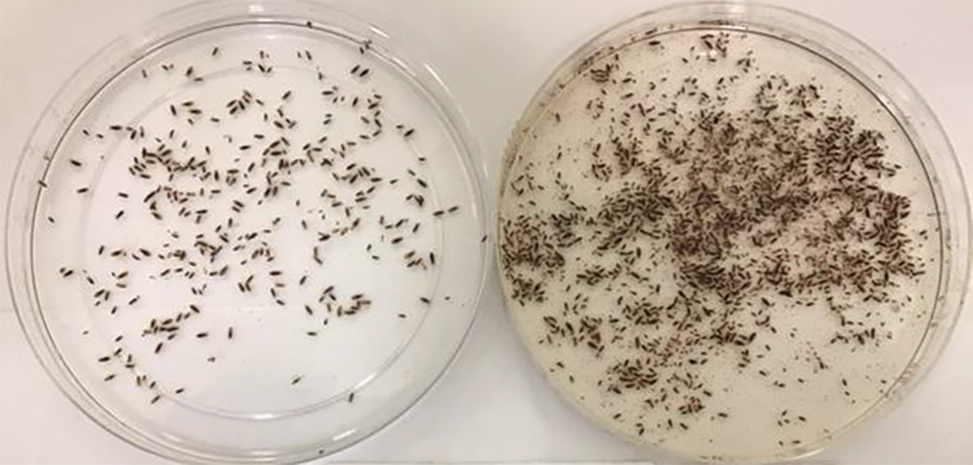
Body lice collected from a person experiencing homelessness in inner city Winnipeg, Manitoba, Canada. Not all ectoparasites from this person were analyzed.

**Figure 2:**
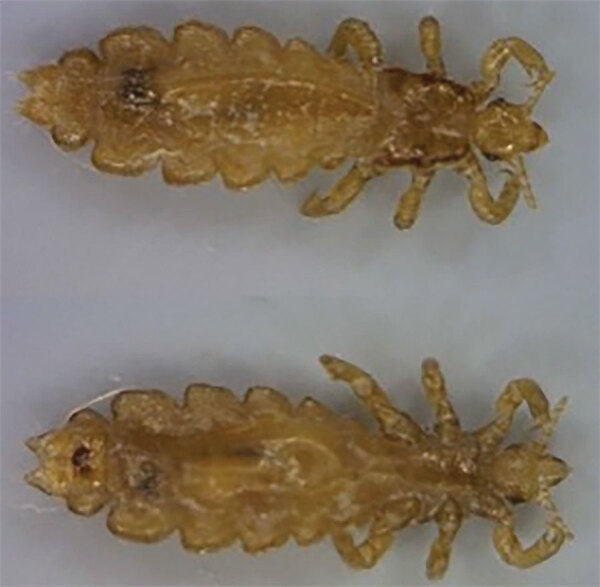
Two female body lice, *Pediculus humanus humanus*, collected from a person experiencing homelessness in inner city Winnipeg, Manitoba, Canada (*9*).

Seven persons submitted ectoparasites, 2 in 2020, and 5 in 2021 (Appendix). We analyzed 556 ectoparasites. The range of ectoparasites tested per participant was 5–218 and per pool was 5–48. We confirmed all ectoparasite pools were *P. humanus humanus* lice by using PCR positivity on louse and body lice targets and morphology (*9*) (Figures 1, 2). All louse pools from 1 participant (1/7 = 14%, 218 lice) demonstrated positivity on all *Bartonella* and *B. quintana* targets (Table 1). Of the 7 louse pools positive for *B. quintana*, 4 also demonstrated molecular positivity for *Acinetobacter* spp. Ectoparasites from all participants were negative for *R. prowazekii* and *C. burnetii*.

**Table 1:** Testing of lice to determine species and infection with *Bartonella quintana* and *Acinetobacter* spp. from a person experiencing homelessness in inner city Winnipeg, Manitoba, Canada*

Pool	No. lice/pool	Instar	Ct values				
			Body louse gene	*Bartonella* ITS3	*B. quintana yopP* gene	*B. quintana fabB* gene	*Acinetobacter rpoB *
1	48	1st and 2nd	31.2	33.6	30.8	30.8	40
2	7	1st and 2nd	29.8	35.6	36.2	35.4	40
3	26	3rd	30.6	25.6	26.2	26.0	36.2
4	26	3rd	30.4	23.0	27.0	26.7	38.5
5	5	3rd	29.9	33.6	34	34.2	40
6	6	3rd	30.5	33.3	34.2	33.8	37.4
7	30	4th	29.3	21.8	23.1	22.9	29.6

*Ct >40 indicates a negative result. Genes used to determine species identities: PHUM540560, body lice gene distinguishing body lice from head lice; ITS3, internal transcribed spacer 3, identifies *Bartonella* to genus level; *yopP*, hypothetical intracellular effector gene, identifies *B. quintana* to species; *fabB*, 3-oxoacyl-synthase gene, identifies *B. quintana* to species; *rpoB*, RNA polymerase β subunit gene, identifies *Acinetobacter* spp. Ct, cycle threshold.

When analyzing *B. quintana–*positive louse pools, we found Ct values were similar between ITS3, *yopP*, and *fabB* genes (test statistic H = 0.54; p = 0.76). The average ITS3 Ct values decreased from the first and second instar pools (34.6) to the third instar pools (28.9) by 5.7, and from the third instar pools to the fourth instar pool (21.8) by 7.1. Pools from female lice demonstrated lower ITS3 Ct values than male lice pools (p = 0.0214) (Table 2).

**Table 2:** Testing of fourth instar body lice pools, divided by sex and associated C) for 3 *Bartonella* genes, from a person experiencing homelessness in Winnipeg, Manitoba, Canada*

Pool	No. lice/pool	Sex	Ct values		
			*Bartonella* ITS3 gene	*B. quintana yopP* gene	*B. quintana fabB* gene
8	6	Female	21.5	23.1	22.7
9	7	Female	24.2	25.2	24.9
10	7	Female	24.6	25.8	25.4
11	7	Female	24.8	25.7	25.5
12	7	Female	25.6	26.6	26.3
13	7	Male	27.2	28.1	27.6
14	7	Male	27.6	27.4	27.2
15	7	Male	25.2	26.1	25.7
16	7	Male	27.6	28.6	28.2
17	8	Male	28.1	29.1	28.6

*Genes used to determine species identities: PHUM540560, body lice gene distinguishing body lice from head lice; ITS3, internal transcribed spacer, identifies *Bartonella* to genus level; *yopP*, hypothetical intracellular effector gene, identifies *B. quintana* to species; *fabB*, 3-oxoacyl-synthase gene, identifies *B. quintana* to species; *rpoB*, RNA polymerase β subunit gene, identifies *Acinetobacter* spp.

## Conclusions

We determined by molecular testing that body lice collected from a person experiencing homelessness in Winnipeg were positive for *B. quintana* bacteria. This finding complements the recent Manitoba cluster of *B. quintana* cases, suggesting a poorly described burden of infection (*1*,*2*,*4*). The hidden presence of *B. quintana* bacteria in Canada was recently highlighted in an outbreak of transplant derived *B. quintana* infection in cities that had not previously reported transmission: 5 cases of bacillary angiomatosis were linked to 3 deceased donors from 2 cities in Alberta (Health Canada, pers. comm., email, 2023 Nov 4). All cases were confirmed to be *B. quintana* bacteria with donors experiencing homelessness as the common risk factor (Health Canada, pers. comm., email, 2023 Nov 4).

Our study suggests a minority of body lice cases from Winnipeg are positive for pathogens, including *B. quintana* bacteria*.* We did not collect epidemiologic data for this study, but all participants were persons who experienced homelessness in inner city Winnipeg. Because of Winnipeg’s harsh winters and few homeless shelters, it is possible the participant with *B. quintana*–positive lice lives in close proximity to others and other persons with *B. quintana* infection remain undocumented. Only 1/7 persons with body lice had *B. quintana*–positive lice, which may be because of the small number of participants and that 3 participants submitted few ectoparasites. Nationwide body lice studies to compare *B. quintana* bacterial prevalence across different areas are needed to identify locations of infection.

The absence of other pathogens likely reflects differences in transmission dynamics and ecology (*10*,*11*). Unlike *B. quintana* bacteria, which does not alter louse survival, lice infected with *R. prowazekii* bacteria die within a week of infection, limiting transmission (*11*). The urban setting of our study diminishes the chance of replicating the occasional documentation of *C. burnetii* bacteria in lice. Whereas *Acinetobacter* spp. bacteria are commonly identified in body lice, no proven cases of *Acinetobacter* disease caused by body lice have been confirmed (*11*,*12*).

The lower *Bartonella* Ct values (stronger signal) with advancing louse instar and female sex may indicate larger blood meals of those subpopulations. *B. quintana* bacteria replicate in the louse intestine but are not known to be transmitted transovarially, indicating the person with *B. quintana*–positive lice from all instars likely had sustained bacteremia for at least 1 month (body lice lifespan). This study highlights the usefulness of identifying ectoparasites by using molecular methods when arthropod taxonomic expertise is not accessible.

*B. quintana* bacteria is excreted in louse feces continuously for weeks in quantities up to 10^7^ bacteria/louse each day (*13*,*14*). The explosive replication, coupled with *B. quintana* bacteria remaining infectious in biofilm-like structures for up to 1 year, suggests even a single case of *B. quintana* infection may indicate a hidden burden of infected persons (*5*,*14*). 

Our study is limited by a small sample size, the heterogenous number of ectoparasites submitted per person, the focus on urban populations from 1 jurisdiction, and the lack of DNA quantity normalization. Active case finding, contact tracing, and public health engagement are needed to clarify the epidemiology of *B. quintana* infection in Canada. Manitoba residents with body lice should be evaluated for *B. quintana* infection. Sampling of ectoparasites may provide an effective way to perform surveillance for emerging pathogens in marginalized settings. 

Appendix Additional information about body louse pathogen surveillance among persons experiencing homelessness, Winnipeg, Canada 2020–2021.
