# Supplementary material for: Body Louse Pathogen Surveillance among Persons Experiencing Homelessness, Canada, 2020–2021
Source: Emerg Infect Dis. 2024 Jul;30(7):1450–3. doi: 10.3201/eid3007.231660 (PMC11210640; doi:10.3201/eid3007.231660)
Supplement: Appendix — Additional information about body louse pathogen surveillance among persons experiencing homelessness, Winnipeg, Canada 2020–2021. [file 23-1660-Techapp-s1.pdf]

Article DOI: http://doi.org/10.3201/eid3007.231660

EID cannot ensure accessibility for supplementary materials supplied by authors. Readers who have difficulty accessing supplementary content should contact the authors for assistance.

Body Louse Pathogen Surveillance Among Persons Experiencing Homelessness, Winnipeg, Canada 2020–2021

Appendix

Real-time PCR procedure and cycling conditions:

We used 5 µL of template DNA in 30 µL reaction volumes containing TaqMan Universal Master Mix (Applied Biosystems). Amplifications were performed on a ViiA7 system (Applied Biosystems) in accordance with manufacturer's instructions. Thermocycling conditions for all targets were 2 min at 50°C, 10 min at 95°C, and 40 cycles of 95°C for 15 s and 60°C for 1 min. We included synthetic DNA (Integrated DNA Technologies, https://www.idtdna.com) of the targets (see table below) as positive controls. Master mix without DNA and sterile deionized water were used as negative controls in each run. A sample was considered positive if cycle threshold (Ct) values were <40. Positive samples were reextracted and retested to ensure reproducibility. Samples with repeated Ct values of <40 were considered positive. We calculated statistics from the initial extraction.

Table 1. Oligonucleotide sequences of primers and probes and references used for real-time PCR

Target	Name	Primers (5′-3′) and probes	Reference
P. humanus, *Cytochrome b*	*Cytb*	F_GAGCGACTGTAATTACTAATC, R_CAACAAAATTATCCGGGTCC, FAM-TAGGAGGCTTTGTGTGTCTATCCT-TAMRA	Li et al. (*1*)
P. humanus humanus, Phum_PHUM540560	Phum	F_GTCACGTTCGACAAATGTT, R_TTTCTATAACCACGACACGATAAAT, FAM-CGATCACTCGAGTGAATTGCCA-TAMRA	Drali et al. (*2*))
***Bartonella* genus**, 16S-23S rRNA intergenic spacer region	ITS3	F_ GGG GAA CCT GTG GCT GGA TCA C, R_ TGAACCTCCGACCTCACGCTTATC, FAM-TTCAGATGATGATCCCAA	Roux et al. (*3*)
Bartonella quintana, Hypothetical intracellular effector	*yopP*	F_TAAACCTCGGGGGAAGCAGA, R_TTTCGTCCTCAACCCCATCA, FAM-CGTTGCCGACAAGACGTCCTTG-TAMRA	Angelakis et al. (*4*)
Bartonella quintana, 3-oxoacyl-synthase	*fabB*	F_GCGGCCTTGCTCTTGATGA, R_GCTACTCTGCGTGCCTTGGA, FAM-TGCAGCAGGTGGAGAGAACGTG-TAMRA	Angelakis et al. (*4*)
Rickettsia prowazekii, Outer membrane protein B	*ompB*	F_AATGCTCTTGCAGCTGGTTCT, R_TCGAGTGCTAATATTTTTGAAGCA, FAM-CGGTGGTGTTAATGCTGCGTTACAACA-TAMRA	Nguyen-Hieu T et al. (*5*)

Target	Name	Primers (5′-3′) and probes	Reference
Coxiella burnetiid, Insertion sequence IS1111	IS1111	F_CAAGAAACGTATCGCTGTGGC, R_CACAGAGCCACCGTATGAATC, FAM-CCGAGTTCGAAACAATGAGGGCTG-TAMRA	Mediannikov et al. (*6*)
***Acinetobacte*r spp.**, RNA polymerase β subunit gene	rpoB	F_TACTCATATACCGAAAAGAAACGG, R_GGYTTACCAAGRCTATACTCAAC, FAM-CGCGAAGATATCGGTCTSCAAGC-TAMRA	Bouvresse et al. (*7*)

Statistical testing:

Statistical testing was performed using R Statistical Software, version 3.5.3, R Foundation for Statistical Computing. The Mann-Whitney U test was used as a non-parametric test to compare Ct values of two groups, with the null hypothesis being that the medians of the two samples are identical. In the manuscript, this test was used to compare Ct values of the ITS3 gene (*Bartonella* species) from male and female 4th instar lice (two groups according to sex). The Kruskal-Wallis Test (Bonferroni correction, post-hoc Dunn's test) was performed as a non-parametric test to compare more than two groups, with the null hypothesis being that the medians of the groups are equal (the post-hoc Dunn’s test, takes into consideration the total number of groups). This test was used to compare the Ct values of the three separate molecular targets associated with *Bartonella quintana* in this study (ITS3, *yopP, fabB,* see table above). *P*-values under 0.05 were considered significant.

Participant eligibility and sampling methodology:

All individuals presenting with pediculosis corporis to one out-patient clinic and one hospital in inner-city Winnipeg, Manitoba, Canada during 2020 and 2021 were eligible to be included in this study. Individuals without visible ectoparasites were excluded, as were individuals who presented to locations outside inner-city Winnipeg.

No sample size calculation occurred before this study. As no previous reports of pediculosis corporis in Canada have been published, it was difficult to estimate the number of participants with pediculosis corporis as well as the number of submitted ectoparasites. All participants meeting eligibility criteria were consented to provide ectoparasites for analysis. All eligible participants during the study time period consented and provided ectoparasites. When hundreds of ectoparasites were collected from a single individual, not all ectoparasites were analyzed.

Institutional ethics review board approval:

The study was approved by the University of Manitoba (H2020:374), the Health Sciences Centre (*RI*2020: 147), St. Boniface Hospital (RRC/2020/1978), Shared Health and Winnipeg Regional Health Authority (2020–059) and the Manitoba Health Information Privacy Committee (2020/2021–79).

Table 2. Ectoparasite number and pools submitted per individual and associated PCR results:

Individual	Year	N ecto	N pool	CytB	Phum	Bquin	OmpB	IS1111
1	2021	218	17	Pos	Pos	Pos	Neg	Neg
2	2021	160	4	Pos	Pos	Neg	Neg	Neg
3	2021	60	3	Pos	Pos	Neg	Neg	Neg
4	2021	90	3	Pos	Pos	Neg	Neg	Neg
5	2021	18	3	Pos	Pos	Neg	Neg	Neg
6	2020	5	1	Pos	Pos	Neg	Neg	Neg
7	2020	5	1	Pos	Pos	Neg	Neg	Neg

N ecto: indicates number of ectoparasites analyzed (N.B. more ectoparasites were collected than analyzed among individuals 1–4). N pool: indicates the number of different pools. CytB: cytochrome B gene targeting *Pediculus humanus* louse PCR result. Phum: indicates positivity for the PHUM540560 body lice gene encoding a protein of unknown function with 22 polymorphisms distinguishing body lice from head lice. B. quin: indicates combined results for ITS3 (internal transcribed spacer gene identifying *Bartonella* to genus-level), *yopP* (hypothetical intracellular effector gene identifying *B. quintana* to species) and *fabB* (3-oxoacyl-synthase gene identifying *B. quintana* to species). Positivity for "B.quin" indicates positivity on all three PCR targets (ITS3, *yopP, fabB*). *ompP*: Outer membrane protein B gene indicating *R. prowazekii.* IS1111: Insertion sequence IS1111 gene indicating *C. burnetii.* Pos: positive PCR result. Neg: negative PCR result.

References

1. Li W, Ortiz G, Fournier PE, Gimenez G, Reed DL, Pittendrigh B, et al. Genotyping of human lice suggests multiple emergencies of body lice from local head louse populations. PLoS Negl Trop Dis. 2010;4:e641. PubMed https://doi.org/10.1371/journal.pntd.0000641

2. Drali R, Boutellis A, Raoult D, Rolain JM, Brouqui P. Distinguishing body lice from head lice by multiplex real-time PCR analysis of the Phum_PHUM540560 gene. PLoS One. 2013;8:e58088. PubMed https://doi.org/10.1371/journal.pone.0058088

3. Roux V, Raoult D. The 16S-23S rRNA intergenic spacer region of *Bartonella* (*Rochalimaea*) species is longer than usually described in other bacteria. Gene. 1995;156:107–11. PubMed https://doi.org/10.1016/0378-1119(94)00919-J

4. Angelakis E, Diatta G, Abdissa A, Trape J-F, Mediannikov O, Richet H, et al. Altitude-dependent *Bartonella quintana* genotype C in head lice, Ethiopia. Emerg Infect Dis. 2011;17:2357–9. PubMed https://doi.org/10.3201/eid1712.110453

5. Nguyen-Hieu T, Aboudharam G, Signoli M, Rigeade C, Drancourt M, Raoult D. Evidence of a louse-borne outbreak involving typhus in Douai, 1710-1712 during the war of Spanish succession. PLoS One. 2010;5:e15405. PubMed https://doi.org/10.1371/journal.pone.0015405

6. Mediannikov O, Fenollar F, Socolovschi C, Diatta G, Bassene H, Molez J-F, et al. *Coxiella burnetii* in humans and ticks in rural Senegal. PLoS Negl Trop Dis. 2010;4:e654. PubMed https://doi.org/10.1371/journal.pntd.0000654

7. Bouvresse S, Socolovshi C, Berdjane Z, Durand R, Izri A, Raoult D, et al. No evidence of *Bartonella quintan*a but detection of *Acinetobacter baumannii* in head lice from elementary schoolchildren in Paris. Comp Immunol Microbiol Infect Dis. 2011;34:475–7. **PMID: 21974965**
